# From voice biomarkers to telemedicine screening: developing and evaluating a voice-based AI model for laryngeal lesion detection using the Bridge2AI-Voice dataset

**DOI:** 10.3389/fdgth.2026.1846369

**Published:** 2026-07-01

**Authors:** Phillip D. Jenkins, Steven Bedrick, Lisa Karstens, William Hersh, the Bridge2AI-Voice Consortium, David A. Dorr

**Affiliations:** 1 Department of Medicine, Division of Informatics and Clinical Epidemiology, Oregon Health & Science University, Portland, OR, United States; 2 Department of Surgery, Surgical Data and Decision Sciences Lab, Oregon Health & Science University, Portland, OR, United States; 3 Biostatistics Shared Resources, Knight Cancer Institute, Oregon Health & Science University, Portland, OR, United States; 4 Division of Oncological Sciences, Knight Cancer Institute, Oregon Health & Science University, Portland, OR, United States

**Keywords:** artificial intelligence, laryngeal neoplasms, machine learning, telemedicine, voice disorders

## Abstract

**Background:**

The human voice contains rich acoustic information indicative of laryngeal pathology, yet current screening relies on resource-intensive in-person laryngoscopy. While artificial intelligence has shown promise for voice analysis, progress has been limited by small, inconsistent datasets and challenges to clinical translation. The Bridge2AI-Voice initiative addresses these barriers by providing a large-scale, ethically sourced dataset with standardized, privacy-preserving derived features.

**Objective:**

To determine whether the derived-feature release of Bridge2AI-Voice v3.0.0 can support a high-sensitivity screening model for laryngeal lesions and to evaluate its translational readiness using telemedicine implementation frameworks.

**Methods:**

We analyzed data from 205 adult participants (136 controls, 52 benign vocal fold lesions, 13 precancerous lesions, 4 laryngeal cancer) drawn from the Bridge2AI-Voice v3.0.0 derived-feature release. An L2-regularized logistic regression model was fit to 131 OpenSMILE static acoustic features with age and sex at birth, evaluated under participant-level stratified 10-fold nested cross-validation. Inner-fold cross-validation was used for operating-point threshold selection. Pre-specified validity tests against age confounding included a DeLong comparison against an age-only baseline and an age-stratified label permutation test. Alternative feature modalities (SPARC articulatory features, Mel spectrogram derivatives, and multimodal combinations) and alternative classifier families were evaluated as robustness checks.

**Results:**

The OpenSMILE-based model achieved cross-validated AUC 0.812 (95% CI 0.744–0.876), with operating-point sensitivity 0.870 (95% CI 0.767–0.939) and specificity 0.566 (95% CI 0.479–0.651). Model discrimination significantly exceeded an age-only baseline (DeLong *p* = 0.0008) and survived age-stratified label permutation (observed AUC 0.812 vs. null mean 0.553, *p* = 0.0099). Subgroup analysis showed approximately consistent sensitivity across benign (0.865) and precancerous (0.846) lesion subgroups. Alternative feature modalities did not provide incremental discriminative information beyond OpenSMILE, and alternative classifier families produced AUCs within bootstrap confidence intervals of the primary model.

**Conclusions:**

Derived acoustic features from the Bridge2AI-Voice v3.0.0 release combined with basic demographic information support cross-validated discrimination of vocal fold lesions consistent with the upper range of published voice-based laryngeal pathology classifiers. The result is presented as a candidate signal warranting confirmatory investigation in a larger, prospectively recruited cohort.

## 1 Background and significance

The human voice is a rich, multidimensional biosignal shaped by the coordinated interaction of respiratory, phonatory, resonatory, and articulatory subsystems. Alterations in these processes can manifest as measurable changes in acoustic and prosodic characteristics, many of which have long been recognized by clinicians as indicators of underlying pathology (1). In otolaryngology, benign and malignant laryngeal lesions modify vocal fold vibration through changes in mass, stiffness, or closure patterns, producing quantifiable variations in parameters such as fundamental frequency (F0), jitter, shimmer, and harmonic-to-noise ratio (HNR) (2–4). These features have been associated with diagnostic and prognostic information, yet traditional clinical evaluation relies on resource-intensive, in-person examinations such as laryngoscopy, which may be limited by access, cost, and delays in care (5). Consequently, there is growing interest in developing scalable, noninvasive, voice-based tools to support earlier detection and triage for conditions that manifest in the acoustic signal (6).

Artificial intelligence (AI) techniques have rapidly expanded the ability to extract clinically relevant information from complex data modalities such as imaging, free text, and physiologic waveforms (7). Similar approaches have recently been applied to voice, demonstrating potential for screening neurological disorders, mood disorders, and voice disorders, including laryngeal cancer (5–7). However, progress has been hindered by several recurring challenges across the literature: small or single-institution datasets, inconsistent recording conditions, limited demographic diversity, incomplete metadata, and a lack of standardized protocols. These limitations restrict reproducibility, external validity, and the translation of voice-AI findings into clinical workflows (8). Prior work from our group on the initial Bridge2AI-Voice v1.1 release applied non-parametric group comparisons (Kruskal–Wallis with Dunn's *post-hoc* and Holm correction) to five canonical acoustic features in a cohort of 176 participants, identifying harmonic-to-noise ratio variability as a candidate distinguishing feature between benign and malignant vocal fold lesions in cisgender men and explicitly motivating subsequent work with a larger cohort, broader feature representation, and classifier-based modeling (6). The present analysis extends that program from descriptive hypothesis generation to a screening-oriented predictive model on the substantially expanded v3.0.0 release.

The Bridge2AI-Voice initiative was established by the National Institutes of Health (NIH) to address these gaps by creating an ethically sourced, multi-institutional, large-scale dataset linking voice recordings to extensive demographic, clinical, and questionnaire-based metadata (9). The v3.0.0 release expands the source cohort to 833 adult participants across five collection sites, introduces precancerous lesion phenotyping, and adds SPARC articulatory features and Mel spectrogram derivatives to the previously available OpenSMILE (10) static features, Praat measures, and spectrogram tensors. The dataset provides standardized derived features extracted using open-source tools while protecting privacy by withholding raw audio under controlled-access mechanisms. This scale and standardization create an unprecedented foundation for advancing voice-based diagnostic research using reproducible computational pipelines.

While prior studies have explored the use of machine learning for voice disorder classification, few have specifically focused on high-sensitivity screening models suitable for telemedicine triage or population outreach (11, 12). Equally few have examined how algorithmic outputs can be embedded into real-world clinical workflows or evaluated using established implementation-science frameworks. To ensure clinical impact, predictive models must not only demonstrate discriminative performance but also be explainable, computationally efficient, and aligned with the organizational, ethical, and sociotechnical requirements of digital health deployment.

In this context, we sought to leverage the Bridge2AI-Voice v3.0.0 dataset to evaluate whether the available derived feature modalities can support cross-validated discrimination of vocal fold lesions in a screening-oriented model, to test the discriminative signal against age confounding, and to situate the model within forward-looking implementation rubrics informed by the Model for Assessment of Telemedicine (MAST) (13), Normalization Process Theory (NPT) (14), and Learning Health System (LHS) (15) principles. We present the work as exploratory rather than as evidence of deployment readiness.

## Objective

Our objective was to evaluate whether the derived-feature release of Bridge2AI-Voice v3.0.0 supports cross-validated discrimination of laryngeal lesions in a screening-oriented model, and to discuss this analysis within forward-looking telemedicine implementation rubrics informed by MAST, NPT, and LHS principles.

## 2 Materials and methods

### 2.1 Data source and cohort construction

The Bridge2AI-Voice v3.0.0 release (9) contained derived-feature recordings from 833 adult participants across five collection sites. Diagnostic phenotyping was harmonized into three target categories aligned with the screening task: laryngeal cancer, benign vocal fold lesions, and precancerous lesions. The control class was defined as participants in the source dataset's designated control file, who by Bridge2AI convention do not carry diagnostic codes for other voice disorders. A small number of lesion-positive participants carried comorbid voice-disorder diagnoses (3 with muscle tension dysphonia, 1 with laryngeal dystonia, 1 with airway stenosis); these were retained in the lesion-positive class because excluding them would have further reduced an already underpowered cohort and because the screening objective is to identify any participant who would benefit from laryngoscopic evaluation. Application of these criteria yielded 224 participants with diagnostic phenotyping. Restriction to participants with derived-feature recordings available across all three feature modalities (OpenSMILE, SPARC, Mel spectrogram) reduced the cohort to 207. Listwise deletion of two participants with missing age yielded the final analytic cohort of 205 participants: 136 controls, 52 benign lesions, 13 precancerous lesions, and 4 laryngeal cancer cases. The lesion-positive class for primary analysis combined the benign, precancerous, and cancer subgroups (*n* = 69), reflecting the clinical screening objective of identifying any laryngoscopy-indicated lesion.

### 2.2 Acoustic feature extraction

The primary feature set comprised 131 OpenSMILE static acoustic features provided in the v3.0.0 derived-feature release, covering fundamental frequency, jitter, shimmer, harmonic-to-noise ratio, MFCC summaries, formant-related variables, loudness measures, and prosodic descriptors. Features with more than 50% missingness or near-zero variance were filtered prior to analysis. Three additional feature modalities were available in the v3.0.0 release and evaluated in modality comparison analyses: SPARC articulatory features (6 features capturing pitch and periodicity statistics), Mel spectrogram derivatives (4 spectral descriptors capturing flatness and energy variability), and combinations thereof.

### 2.3 Participant-level aggregation

Because participants contributed multiple recordings across heterogeneous speech tasks, features were aggregated to a single vector per participant using within-participant median across all valid recordings. This approach minimized the influence of outliers, variable recording conditions, and task-specific pauses, and allowed the model to reflect stable participant-level voice characteristics rather than task-dependent fluctuations. Within-fold median imputation handled the small number of missing feature values; within-fold z-score standardization was applied to all continuous features. Both imputation and standardization were applied via a scikit-learn Pipeline to ensure all preprocessing was fit on training-fold data only, preventing leakage.

### 2.4 Univariate feature comparisons

Group-level distributions of acoustic features were compared across diagnostic groups using Kruskal–Wallis tests. Bonferroni correction was applied within each feature class (five canonical acoustic features; four spectrogram-derived features). Five canonical features were pre-specified: mean F0 semitone, mean HNR, HNR variability, mean jitter, and mean shimmer. Four spectrogram-derived spectral descriptors were also tested: mean spectral flatness, standard deviation of flatness, energy variance, and energy standard deviation.

### 2.5 Primary model and cross-validation

The primary model was an L2-regularized logistic regression fit to the 131 OpenSMILE static features with age (continuous) and sex at birth (binary) as additional covariates (133 features total). Regularization strength was fixed at the scikit-learn default (C = 1.0); inner cross-validation was used for operating-point threshold selection only. The model was evaluated under participant-level stratified 10-fold outer cross-validation. Within each outer fold, a 10-fold inner cross-validation on the outer training partition identified the highest probability cutoff at which inner-CV sensitivity was at least 0.85, an *a priori* target selected to favor high-sensitivity operating points appropriate for a triage tool intended to identify candidates for further laryngoscopic evaluation, where the follow-up test (laryngoscopy) is itself a relatively low-risk procedure. The selected threshold was applied to outer-fold predictions to produce per-fold confusion matrix counts. We pre-specified that we would report cross-validated AUC as the primary endpoint and provide operating-point sensitivity, specificity, and confusion matrix counts as supporting evidence. Bootstrap 95% confidence intervals on AUC were computed from 1,000 resamples of the out-of-fold predictions. Clopper-Pearson exact binomial confidence intervals were computed on the aggregated outer-fold confusion matrix for sensitivity and specificity.

### 2.6 Validity tests against demographic confounding

Two pre-specified validity tests were performed to assess whether the observed discrimination reflects age confounding rather than acoustic information. First, an age-only baseline logistic regression was fit and evaluated under the same 10-fold cross-validation scheme as the primary model. The primary model's AUC was compared to the age-only baseline's AUC using DeLong's test on paired out-of-fold predictions. Second, an age-stratified label permutation test was performed: lesion-status labels were permuted within age-decile strata across 100 iterations, and the primary model was re-evaluated under cross-validation at each iteration to generate an empirical null distribution of AUC values. The observed AUC was compared to this null distribution to compute a one-sided empirical *p*-value.

### 2.7 Feature modality comparison and robustness checks

To assess whether feature modalities beyond OpenSMILE static features contributed to discriminative performance, the same nested cross-validation procedure was applied to five additional feature configurations on the same *n* = 205 cohort: SPARC features alone, Mel spectrogram derivatives alone, OpenSMILE combined with SPARC, OpenSMILE combined with Mel-derived features, and the combination of all three modalities. Each configuration was augmented with age and sex at birth covariates. To assess whether the primary model architecture was idiosyncratic, alternative classifier families (elastic net via LogisticRegressionCV, support vector machine with RBF kernel, random forest, Gaussian Naive Bayes, k-nearest neighbors) were evaluated on the OpenSMILE feature space under 10-fold cross-validation as a robustness check; full hyperparameter specifications are provided in Supplementary Table S1 and results in Supplementary Table S2.

### 2.8 Ethical and privacy considerations

Because the dataset contains sensitive voice-derived biomedical information, all analyses were conducted on de-identified derived features in accordance with the Bridge2AI-Voice data-use agreement. No raw audio was accessed. Model construction emphasized interpretability, minimized feature leakage, and maintained alignment with ethical guidelines for machine learning in health care.

## 3 Results

### 3.1 Cohort

The analytic cohort comprised 205 participants drawn from the Bridge2AI-Voice v3.0.0 release: 136 lesion-negative controls and 69 lesion-positive participants (52 benign lesions, 13 precancerous lesions, 4 laryngeal cancer). Demographic characteristics by diagnostic group are summarized in Table 1. Lesion-positive participants were older than controls (median 55.0 vs. 39.0 years), consistent with the known age distribution of laryngeal lesions and motivating the formal age-confounding analyses described below.

**Table 1:** Demographics by diagnostic group (*n* = 205 analytic cohort).

Characteristic	Control (*n* = 136)	Benign (*n* = 52)	Precancerous (*n* = 13)	Cancer (*n* = 4)
Age, years				
Mea*n* ± SD	44.3 ± 19.4	53.3 ± 16.1	65.0 ± 14.7	70.8 ± 5.1
Median (IQR)	39.0 (27.0–63.0)	53.5 (41.8–67.2)	69.0 (59.0–76.0)	71.0 (67.2–74.5)
Sex at birth, *n* (%)				
Female	71 (52%)	29 (56%)	2 (15%)	0 (0%)
Male	64 (47%)	22 (42%)	10 (77%)	4 (100%)

Sex at birth was missing for 3 participants (1 control, 1 benign, 1 precancerous). Percentages reflect proportion of participants with non-missing values in each diagnostic group.

### 3.2 Univariate acoustic feature comparisons

Group-level distributions of canonical acoustic features were compared across diagnostic groups using Kruskal–Wallis tests with Bonferroni correction. None of the five canonical features reached statistical significance after Bonferroni correction (smallest Bonferroni-corrected *p* = 0.060 for HNR variability). The four spectrogram-derived spectral descriptors also did not reach significance after Bonferroni correction at this sample size (smallest Bonferroni-corrected *p* = 0.557 for mean spectral flatness). Univariate comparisons of canonical features are summarized in Table 2. The absence of significant univariate group differences after multiplicity correction indicates that the discriminative signal in this cohort, if present, is multivariate rather than concentrated in any single canonical or spectrogram-derived measure.

**Table 2:** Kruskal–wallis tests comparing canonical acoustic features across diagnostic groups (*n* = 205 analytic cohort).

Feature	H statistic	Raw *p*	Bonferroni-corrected *p*
HNR variability	10.95	0.012	0.060
Jitter (mean local)	9.48	0.024	0.118
F0 semitone (mean)	7.58	0.056	0.278
Shimmer (mean local)	4.36	0.226	1.000
HNR (mean)	2.77	0.429	1.000

### 3.3 Primary model performance

**Figure 1:**
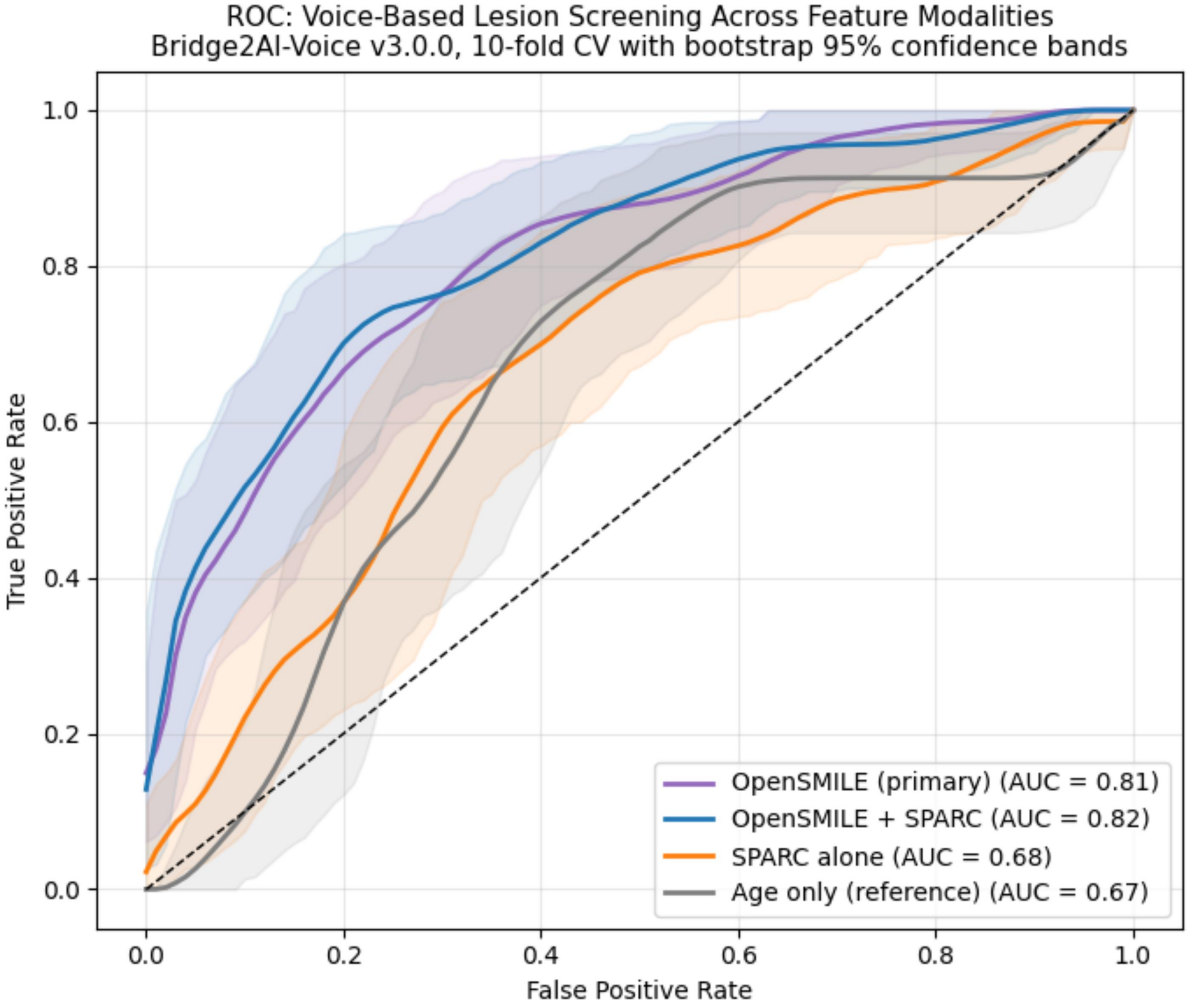
Receiver operating characteristic curves for voice-based laryngeal lesion screening on the Bridge2AI-Voice v3.0.0 analytic cohort (*n* = 205). Curves shown reflect the primary model (L2-regularized logistic regression on OpenSMILE static features with age and sex at birth, AUC 0.812), a multimodal extension adding SPARC articulatory features (AUC 0.817), a single-modality comparison using SPARC features alone (AUC 0.676), and an age-only reference baseline (AUC 0.667). Shaded bands indicate bootstrap 95% confidence intervals. The primary model significantly exceeded the age-only baseline (DeLong *p* = 0.0008), while the SPARC-alone configuration did not (DeLong *p* = 0.749). The OpenSMILE + SPARC multimodal configuration achieved an AUC within the bootstrap confidence interval of the primary model, indicating that adding the SPARC articulatory features did not provide incremental discriminative information beyond the OpenSMILE feature set. All curves reflect aggregated out-of-fold predictions across 10-fold nested cross-validation.

The primary L2-regularized logistic regression model fit to the OpenSMILE static feature set (131 features) with age and sex at birth (133 total features), evaluated under participant-level stratified 10-fold nested cross-validation, achieved a cross-validated area under the receiver operating characteristic curve of 0.812 (95% bootstrap CI 0.744–0.876). At the inner-CV-selected operating point, the model achieved sensitivity 0.870 (60/69 lesion-positive participants identified; 95% Clopper-Pearson CI 0.767–0.939) and specificity 0.566 (77/136 controls correctly classified; 95% CI 0.479–0.651). The confusion matrix at the operating point comprised 60 true positives, 9 false negatives, 77 true negatives, and 59 false positives. The receiver operating characteristic curve for the primary model, alongside an age-only reference baseline and selected feature-modality comparisons, is shown in Figure 1.

### 3.4 Validity against demographic confounding

To address the concern that observed discrimination might be attributable to age confounding rather than acoustic information, two pre-specified validity tests were performed. An age-only baseline logistic regression, evaluated under the same cross-validation scheme, achieved AUC 0.667 (95% CI 0.595–0.744). The primary model's AUC of 0.812 significantly exceeded this baseline (DeLong test, z = 3.34, *p* = 0.0008). In an age-stratified label permutation test, outcome labels were permuted within age-decile strata across 100 iterations. The observed AUC of 0.812 substantially exceeded the empirical null distribution (null mean AUC 0.553), with one-sided empirical *p* = 0.0099. These results support that the discriminative signal captured by the primary model is not attributable to age confounding alone.

### 3.5 Feature modality comparison

To assess whether feature modalities beyond OpenSMILE static features contributed to discriminative performance, the same nested cross-validation pipeline was applied to five additional feature configurations. Results are summarized in Table 3.

**Table 3:** Cross-validated performance across feature modalities (*n* = 205; L2-regularized logistic regression; 10-fold nested CV).

Feature modality	Features	AUC (95% CI)	Sensitivity (95% CI)	Specificity (95% CI)	DeLong *p*
OpenSMILE (primary)	133	0.812 (0.744–0.876)	0.870 (0.767–0.939)	0.566 (0.479–0.651)	0.0008
OpenSMILE + SPARC	139	0.817 (0.750–0.880)	0.841 (0.733–0.918)	0.581 (0.493–0.665)	0.0006
OpenSMILE + SPARC + Mel	143	0.808 (0.740–0.872)	0.841 (0.733–0.918)	0.581 (0.493–0.665)	0.0013
OpenSMILE + Mel	137	0.805 (0.738–0.870)	0.841 (0.733–0.918)	0.559 (0.471–0.644)	0.0016
SPARC alone	8	0.676 (0.602–0.753)	0.841 (0.733–0.918)	0.382 (0.300–0.470)	0.749
Mel-derived alone	6	0.662 (0.590–0.741)	0.841 (0.733–0.918)	0.426 (0.342–0.514)	0.740

All configurations include age and sex at birth as covariates; the Features column reports the total feature count including these two demographic covariates (e.g., the OpenSMILE primary model comprises 131 OpenSMILE features plus age and sex = 133). AUC CIs are bootstrap (1,000 resamples); sensitivity and specificity CIs are Clopper-Pearson; operating points reflect inner-CV threshold selection at target sensitivity 0.85. DeLong *p*-values compare each configuration against the age-only baseline (AUC 0.667).

Among the six feature modality configurations, the four OpenSMILE-containing configurations clustered tightly between AUC 0.805 and 0.817 with overlapping confidence intervals; differences among them are within bootstrap variation. Configurations not including OpenSMILE features (SPARC alone, Mel-derived alone) produced lower AUC and did not exceed the age-only baseline (DeLong *p* = 0.749 and *p* = 0.740). Multimodal combinations of OpenSMILE with SPARC or Mel features did not improve discrimination beyond OpenSMILE alone. These results indicate that for the present cohort and feature representations, the OpenSMILE static feature set captured the available discriminative signal; additional modality concatenation did not contribute incremental information.

### 3.6 Subgroup performance

Sensitivity was approximately equivalent across the benign and precancerous lesion subgroups (0.865 and 0.846 respectively; Table 4). All four laryngeal cancer participants were correctly classified, although the laryngeal cancer subgroup is too small (*n* = 4) for this estimate to be informative; the 95% confidence interval spans 0.40–1.00. The consistency of subgroup-level sensitivity across the benign, precancerous, and cancer categories supports treating the composite lesion-positive class as a coherent screening target rather than reporting separate models per subtype, though the cancer subgroup remains underpowered.

**Table 4:** Lesion-subtype-specific sensitivity for the primary model (*n* = 205 analytic cohort).

Subgroup	*n*	Correctly identified	Sensitivity	95% Clopper-Pearson CI
Benign lesion	52	45	0.865	0.742–0.944
Precancerous lesion	13	11	0.846	0.546–0.981
Laryngeal cancer	4	4	1.000	0.398–1.000
**Total lesion-positive**	**69**	**60**	**0**.**870**	**0.767–0.939**

Bold values indicate the aggregate (composite lesion-positive) result across all lesion subtypes.

### 3.7 Summary of key findings

In an exploratory analysis of the Bridge2AI-Voice v3.0.0 release, an L2-regularized logistic regression model fit to the OpenSMILE static feature set with age and sex at birth achieved cross-validated AUC 0.812 (95% CI 0.744–0.876) for distinguishing participants with vocal fold lesions from controls, with operating-point sensitivity 0.870 and specificity 0.566. The model's discrimination significantly exceeded an age-only baseline (DeLong *p* = 0.0008) and survived age-stratified label permutation (*p* = 0.0099), supporting that the captured signal is not attributable to age confounding alone. Comparison across feature modalities indicated that the OpenSMILE feature set captured the available discriminative signal; SPARC articulatory features and Mel spectrogram derivatives did not exceed age alone as standalone modalities, and multimodal combinations did not improve discrimination beyond OpenSMILE.

## 4 Discussion

### 4.1 Principal findings

In an exploratory analysis of the Bridge2AI-Voice v3.0.0 release, a regularized logistic regression model on standardized acoustic features combined with age and sex at birth achieved cross-validated AUC 0.812 for distinguishing participants with vocal fold lesions from controls, with operating-point sensitivity 0.870 and specificity 0.566 at a triage-oriented threshold. Discrimination significantly exceeded an age-only baseline and survived age-stratified label permutation, supporting that the captured signal is not attributable to age confounding alone. Comparison across feature modalities indicated that the OpenSMILE static feature set captured the available discriminative signal; additional modalities (SPARC articulatory features, Mel spectrogram derivatives, and their multimodal combinations) did not provide incremental information at this cohort scale. Subgroup analysis showed approximately consistent sensitivity across benign and precancerous lesion categories.

### 4.2 Comparison to published voice-based laryngeal pathology models

The cross-validated AUC of 0.812 and operating-point sensitivity of 0.870 achieved in this analysis fall within the range of voice-based machine learning approaches to laryngeal pathology in the published literature, though direct comparison across studies is constrained by substantial methodological heterogeneity in classification task, dataset, and feature configuration. The most directly comparable benchmark is Paterson et al. (16), who evaluated a benchmark suite of voice-based models for binary classification of benign vs. malignant vocal fold pathologies using comparable OpenSMILE features and demographic covariates. Their best voice-plus-demographics model, also an L2-regularized logistic regression on OpenSMILE features, achieved AUC 0.836, sensitivity 0.880, and specificity 0.715 on a holdout test set (*n* = 635), with external sensitivity 0.842 and specificity 0.652 on an independent test set (*n* = 1,295). Other voice-based ML studies in laryngeal pathology differ in task structure: Hu et al. (17) reported sensitivity 0.99 for binary classification of any pathological voice vs. normal voice using a convolutional neural network on Mandarin voice data, but included neurological and atrophic conditions in the pathological class; Marchese et al. (18) reported up to 85% accuracy for polyp identification in males but as part of a 4-class differential diagnosis among benign lesion subtypes without healthy controls; Low et al. (19) reported AUC 0.83–0.88 across four classifier families for binary unilateral vocal fold paralysis vs. healthy classification, though paralysis was excluded from the lesion-positive class in the present analysis.

Notably, the architecture used in the present analysis, an L2-regularized logistic regression on 131 OpenSMILE features, is among the lightest-weight approaches in this literature. Paterson et al. explicitly compared support vector machines, multilayer perceptrons, and logistic regression with this feature set and concluded that classical machine learning algorithms combined with feature extraction matched or outperformed deep learning approaches in their cohort. Low et al. similarly observed that AUCs across logistic regression, stochastic gradient descent, random forest, and multilayer perceptron classifiers clustered tightly together, with no meaningful advantage to the more complex models. Hu et al. achieved high performance using a convolutional neural network with transfer learning from ImageNet and ensemble methods, but at substantially greater architectural complexity and computational cost (17). The convergence of evidence across these studies suggests that for voice-based laryngeal pathology screening at present dataset scales, feature-engineered regularized linear models on standardized acoustic feature sets provide a favorable balance of discriminative performance, interpretability, and deployment feasibility. The model presented here is consistent with this pattern: a parsimonious classifier with full feature-level interpretability, capable of inference on commodity hardware in milliseconds, with no requirement for raw audio retention beyond the feature extraction step.

Low et al. further emphasize that voice-based ML models in this domain are vulnerable to recording-related confounds including audio duration and intensity bias, which they argue must be explicitly addressed (19). We mitigated analogous concerns in the present analysis through within-fold standardization, an age-only baseline comparison, and age-stratified label permutation testing.

### 4.3 Clinical role and screening context

The clinical role envisioned for a voice-based lesion screening tool is upstream of laryngoscopy, not as a replacement for it. The 2018 American Academy of Otolaryngology clinical practice guideline for hoarseness recommends laryngoscopic examination when dysphonia persists for more than four weeks (20). In practice, however, laryngoscopy is invasive, requires specialist equipment, and is geographically and economically inaccessible to many patients. Choi et al. (5) documented the feasibility and limitations of telemedicine-based laryngology evaluation. A voice-based screening tool deployed at the primary care interface or through a patient-facing application could identify which patients with persistent dysphonia should be prioritized for in-person laryngoscopic evaluation, particularly in settings where laryngology access is limited.

At the operating point evaluated in the present analysis, the model identified 60 of 69 lesion-positive participants (sensitivity 0.870) at the cost of flagging 59 of 136 controls as scope-indicated (specificity 0.566). In a screening context, this trade-off favors avoidance of missed lesions over avoidance of unnecessary follow-up evaluations. The clinical defensibility of this trade-off depends on the downstream test: laryngoscopy is uncomfortable but low-risk, and the consequences of missing a benign lesion are typically modest, while missing a precancerous or malignant lesion has more serious implications. The composite lesion-positive class used in the present analysis was constructed to support this triage logic, with the model designed to identify any laryngoscopy-indicated lesion rather than to distinguish lesion subtypes.

Beyond the specific task of vocal fold lesion screening, this work illustrates that derived-feature datasets can support development of clinical AI tools even when access to raw biomedical signals is restricted. Privacy constraints often limit the ability to distribute raw recordings, images, or physiological waveforms; the present findings demonstrate that when derived representations are sufficiently expressive, clinically relevant signals can still emerge. This has implications for the design of future large-scale datasets that aim to balance privacy, accessibility, and clinical utility. The telemedicine framing of this work also highlights potential opportunities to address disparities in access to specialty laryngology care. Rural regions and underserved populations face barriers to timely evaluation of hoarseness and other symptoms of laryngeal disease. A remote voice-based screening tool, embedded in a patient portal or conversational interface, could in principle reduce the need for in-person pre-evaluation and shorten time to diagnosis, particularly if paired with multilingual voice tasks and accessibility features. We frame these access implications as motivating directions for confirmatory work, not as claims that the present analysis establishes any such impact.

The cancer subgroup in this analysis is too small (*n* = 4) to support inference about cancer-specific sensitivity, and we explicitly do not claim that this model is validated for cancer detection. Future work on a substantially larger and oversampled-for-cancer cohort would be required to make any clinically meaningful claim about cancer-specific screening performance.

### 4.4 Implementation considerations and forward-looking rubrics

The forward translation of any screening tool from analytic performance to clinical implementation depends on factors beyond cross-validated discrimination. Established implementation frameworks, including the Model for Assessment of Telemedicine (MAST) (13), Normalization Process Theory (NPT) (14), and Learning Health System (LHS) principles (15), provide structured rubrics for evaluating these factors. We do not claim that the present analysis satisfies the criteria within these frameworks for clinical deployment; rather, we use the frameworks here to identify what would need to be established in subsequent confirmatory work.

Within the MAST framework, the present analysis addresses clinical effectiveness only at the level of analytic discrimination on retrospective data; safety, organizational impact, and patient experience domains are unaddressed. Within NPT, the interpretability and computational lightness of the present model support coherence (a clear behavioral logic for end users) and collective action (feasible integration into existing primary care workflows), but the cognitive participation and reflexive monitoring domains require prospective deployment data that this analysis cannot provide. LHS principles favor model architectures that support iterative recalibration as new data accrue; the regularized logistic regression model used here can be retrained on demand, but no recalibration infrastructure currently exists. These framework considerations are intended to motivate the design of subsequent confirmatory studies, not to claim that the present analysis demonstrates deployment readiness.

### 4.5 Regulatory pathway considerations

Should development continue past the exploratory analysis presented here, a voice-based screening tool of this type would meet the FDA definition of Software as a Medical Device (SaMD). Under the 2017 IMDRF SaMD risk categorization framework, a tool that informs clinical management decisions about non-life-threatening conditions falls in a moderate-risk SaMD category. The development pathway would require, at minimum, prospective validation on an independent cohort, demonstrated generalizability across recording environments and patient demographics, and a clinical decision algorithm that integrates model output with clinical context rather than functioning as an autonomous diagnostic. The present analysis is not positioned to meet any of these standards; we present it as evidence that the discriminative signal warrants further investigation, not as a candidate for regulatory submission.

### 4.6 Limitations

Several limitations bear on the interpretation of these results. First, the analytic cohort (*n* = 205) is modest, with particular underrepresentation of the laryngeal cancer subgroup (*n* = 4). The 95% confidence intervals on operating-point sensitivity (0.767–0.939) and specificity (0.479–0.651) are correspondingly wide, and the laryngeal cancer sensitivity estimate is essentially uninformative at this sample size.

Second, the Bridge2AI-Voice cohort is a multi-site research collection with standardized recording protocols. Generalization to recordings made on consumer devices in uncontrolled acoustic environments cannot be assumed and would require explicit out-of-distribution validation. Low et al. (19) demonstrated that voice-based ML models in this domain are vulnerable to recording-protocol biases including audio duration and microphone gain effects; while our within-fold standardization and age-stratified permutation testing address some of these concerns, the deployment environment for any future voice-based screening application is likely to differ substantially from the research-grade recording conditions under which the training data were collected.

Third, the operating point reported here was selected to favor high sensitivity, with specificity (0.566) only modestly above chance. Whether this trade-off is clinically defensible depends on the population prevalence of laryngeal lesions, the cost and risk of follow-up laryngoscopy, and the downstream consequences of false negatives across lesion subtypes; these parameters are not estimated within the present analysis.

Fourth, the lesion-positive class combines benign, precancerous, and laryngeal cancer subgroups. While the consistency of subgroup-level sensitivities supports treating these as a coherent triage target, the model has not been demonstrated to provide subtype-specific information, and any clinical interpretation should treat the model output as a binary scope-indicated/not-scope-indicated flag rather than as a probability of malignancy.

Fifth, the present analysis used only features derived from the standardized Bridge2AI-Voice release; no raw audio processing was performed. While this design supports privacy-preserving deployment and is consistent with the ethical framework of the source dataset, it also forecloses analyses based on alternative feature representations including more recent self-supervised audio embedding models. Future work could evaluate whether such embeddings provide incremental discriminative information beyond the OpenSMILE static features used here.

### 4.7 Conclusion

This exploratory analysis demonstrates that derived acoustic features from the Bridge2AI-Voice v3.0.0 release, when combined with basic demographic information in a regularized logistic regression model, achieve cross-validated discrimination of vocal fold lesions from controls (AUC 0.812) that is consistent with the upper range of published voice-based laryngeal pathology classifiers and that is not attributable to age confounding alone. The model is computationally lightweight, fully interpretable, and architecturally consistent with current evidence that classical regularized models on standardized acoustic feature sets perform competitively with deep learning approaches in this domain at present dataset scales. The result is presented as a candidate signal warranting confirmatory investigation in a larger, prospectively recruited cohort with the deployment-environment characteristics required to support the regulatory and implementation pathways described above. The framework considerations and limitations articulated here are intended to scaffold subsequent work, not to claim present deployment readiness.

## Data Availability

Publicly available datasets were analyzed in this study. This data can be found here: https://physionet.org/content/b2ai-voice/3.0.0/.

## Group members of the Bridge2AI-Voice Consortium

Group members of the Bridge2AI-Voice Consortium (version 3.0.0 order): Yael Bensoussan, Alexandros Sigaras, Anais Rameau, Olivier Elemento, Maria Powell, David Dorr, Philip Payne, Vardit Ravitsky, Jean-Christophe Bélisle-Pipon, Ruth Bahr, Stephanie Watts, Donald Bolser, Jennifer Siu, Jordan Lerner-Ellis, Frank Rudzicz, Micah Boyer, Yassmeen Abdel-Aty, Toufeeq Ahmed Syed, James Anibal, Dona Amraei, Stephen Aradi, Kirollos Armosh, Ana Sophia Martinez, Shaheen Awan, Steven Bedrick, Helena Beltran, Alexander Bernier, Moroni Berrios, Isaac Bevers, Alden Blatter, Rahul Brito, Amy Brown, Johnathan Brown, Léo Cadillac, Selina Casalino, John Costello, Abhijeet Dalal, Iris De Santiago, Enrique Diaz-Ocampo, Amanda Doherty-Kirby, Mohamed Ebraheem, Ellie Eiseman, Mahmoud Elmahdy, Renee English, Emily Evangelista, Kenneth Fletcher, Hortense Gallois, Gaelyn Garrett, Alexander Gelbard, Anna Goldenberg, Karim Hanna, William Hersh, Jennifer Jain, Lochana Jayachandran, Kaley Jenney, Kathy Jenkins, Stacy Jo, Alistair Johnson, Ayush Kalia, Megha Kalia, Zoha Khawa, Cindy Kostelnik, Alisa Krause, Andrea Krussel, Elisa Lapadula, Genelle Leo, Justin Levinsky, Chloe Loewith, Radhika Mahajan, Vrishni Maharaj, Siyu Miao, LeAnn Michaels, Matthew Mifsud, Marian Mikhael, Elijah Moothedan, Yosef Nafii, Tempestt Neal, Karlee Newberry, Evan Ng, Christopher Nickel, Amanda Peltier, Trevor Pharr, Michaela Pnacekova, Matthew Pontell, Claire Premi-Bortolotto, Parnaz Rafatjou, JM Rahman, John Ramos, Sarah Rohde, Michael de Riesthal, Jillian Rossi, Laurie Russell, Samantha Salvi Cruz, Joyce Samuel, Suketu Shah, Ahmed Shawkat, Elizabeth Silberholz, John Stark, Lala Su, Shrramana Ganesh Sudhakar, Duncan Sutherland, Venkata Swarna Mukhi, Jeffrey Tang, Luka Taylor, Jamie Toghranegar, Julie Tu, Megan Urbano, Gavin Victor, Kimberly Vinson, Jordan Wilke, Claire Wilson, Madeleine Zanin, Xijie Zeng, Theresa Zesiewicz, Robin Zhao, Pantelis Zisimopoulos, Satrajit Ghosh.
