# Supplementary material for: From voice biomarkers to telemedicine screening: developing and evaluating a voice-based AI model for laryngeal lesion detection using the Bridge2AI-Voice dataset
Source: Front Digit Health. 2026 Jul 1;8:1846369. doi: 10.3389/fdgth.2026.1846369 (PMC13368934; doi:10.3389/fdgth.2026.1846369)
Supplement: Supplementary file 1 [file Datasheet1.pdf]

Supplementary Table S1. Hyperparameters and procedural specifications.

Parameter	Value
Primary model: L2-regularized logistic regression	
Feature set	131 OpenSMILE static features + age + sex at birth (133 total)
Regularization	L2 (Ridge)
Regularization strength (C)	1.0 (fixed; not tuned via inner CV)
Solver	lbfgs (scikit-learn default)
Maximum iterations	1000
Class weighting	None
Feature standardization	Within-fold z-score (sklearn StandardScaler in Pipeline)
Missing-data handling	Within-fold median imputation (sklearn SimpleImputer in Pipeline)
Nested cross-validation procedure	
Outer CV	StratifiedKFold, 10 splits, shuffle=True, random_state=42
Inner CV (threshold selection only)	StratifiedKFold, 10 splits, shuffle=True, random_state=42 + fold_idx
Threshold selection rule	Lowest probability cutoff at which inner-CV true-positive rate ≥ 0.85
Bootstrap confidence intervals	
AUC	1000 bootstrap resamples of OOF predictions, 2.5th and 97.5th percentiles, random_state=42
Sensitivity / specificity	Clopper-Pearson exact binomial on aggregated outer-fold confusion matrix
Validity tests	
Age-only baseline	Logistic regression with age only, max_iter=1000, 10-fold CV with random_state=42
DeLong test	Two-sided, paired on common participant IDs
Age-stratified permutation	100 iterations, labels permuted within age-decile strata
Alternative classifiers (Supplementary Table S2)	
Elastic Net	LogisticRegressionCV, penalty='elasticnet', solver='saga', Cs=10, l1_ratios=[0.1, 0.5, 0.9], cv=5, max_iter=5000, scoring='roc_auc'
Support Vector Machine	SVC, kernel='rbf', C=1.0, gamma='scale', probability=True

Random Forest	RandomForestClassifier, n_estimators=200, max_depth=None, min_samples_leaf=1
Gaussian Naive Bayes	GaussianNB (scikit-learn defaults)
K-Nearest Neighbors	KNeighborsClassifier, n_neighbors=5, weights='uniform'

Supplementary Table S2. Alternative classifier performance under cross-validation.

Alternative classifiers were evaluated on the OpenSMILE feature set (133 features) with age and sex at birth covariates, using 10-fold cross-validation on the full OpenSMILE cohort (n=224) as a methodological robustness check on the OpenSMILE feature space. The primary model in main-text Table 3 (L2 logistic on the n=205 modality-intersection cohort with nested CV for threshold selection, AUC 0.812, 95% CI 0.744-0.876) is reported on the more restrictive cohort common to all three feature modalities; the alternative classifiers here are reported on the larger n=224 cohort because they were evaluated as a methodological robustness check rather than as part of the modality comparison. Alternative classifiers cluster within bootstrap confidence intervals of the primary model.

Classifier	AUC	95% CI
Logistic Regression (L2)	0.832	0.773-0.887
Elastic Net	0.817	0.753-0.877
Support Vector Machine (RBF kernel)	0.816	0.754-0.872
Random Forest	0.793	0.728-0.853
Gaussian Naive Bayes	0.748	0.678-0.813
K-Nearest Neighbors	0.747	0.683-0.808

Confidence intervals are 1000-bootstrap resamples of out-of-fold predictions.
